# Gastric Type 1 Neuroendocrine Tumor in an Elderly Patient: A Case Report and Diagnostic Approach Review

**DOI:** 10.7759/cureus.87429

**Published:** 2025-07-07

**Authors:** Emmanuel E Cortés-Marín, José C González-Rodríguez, Maria Cristofori, José A Antunez Oliva, Maria F Vargas Wille

**Affiliations:** 1 Internal Medicine, Universidad de Costa Rica, San José, CRI; 2 General Practice, Universidad de Ciencias Médicas, San José, CRI; 3 Pathology, Universidad de Costa Rica, San Jose, CRI

**Keywords:** anemia, atrophic gastritis, elderly, gastric neuroendocrine tumor, hypergastrinemia

## Abstract

Gastric neuroendocrine tumors (G-NETs) are rare neoplasms with increasing incidence due to the broader use of endoscopy and improved diagnostic sensitivity. Type 1 G-NETs are the most common subtype and are typically associated with chronic atrophic autoimmune gastritis and hypergastrinemia. We present the case of an 84-year-old female patient admitted for the evaluation of progressive fatigue, weight loss, anorexia, and intermittent diarrhea. Initial workup revealed severe iron-deficiency anemia. Further testing demonstrated a positive interferon-gamma release assay (IGRA), with no evidence of active tuberculosis. Colonoscopy and gynecologic evaluation ruled out colorectal and adnexal malignancies. Esophagogastroduodenoscopy identified multiple raised vascular lesions in the gastric fundus and body. Histopathology confirmed a well-differentiated type 1 G-NET (grade 2, Ki-67 index 5%), associated with enterochromaffin-like cell hyperplasia and autoimmune atrophic gastritis. The patient was managed through multidisciplinary evaluation. Given the small size, low grade, and absence of metastasis, a conservative approach with endoscopic surveillance was chosen. While endoscopic resection or surgical interventions such as antrectomy or gastrectomy may be appropriate in selected cases, functional assessment and individualized decision-making remain essential, particularly in older adults with multimorbidity. This case highlights the diagnostic complexity of type 1 G-NETs in elderly patients and underscores the importance of tailored, multidisciplinary management strategies.

## Introduction

Gastric neuroendocrine tumors (G-NETs) are an infrequent clinicopathological entity whose incidence has increased in recent decades, partly due to the widespread use of endoscopy and greater diagnostic awareness. They account for 1% to 2% of gastric neoplasms and arise from enterochromaffin-like (ECL) cells located in the fundus and gastric body [1].

Clinically and pathophysiologically, G-NETs are categorized into three main subtypes. Type 1, which comprises 70%-80% of cases, is associated with chronic autoimmune atrophic gastritis, hypergastrinemia, and ECL cell hyperplasia [2]. Type 2 is linked to Zollinger-Ellison syndrome in the context of multiple endocrine neoplasia type 1 (MEN-1), while type 3 corresponds to sporadic, gastrin-independent tumors with a more aggressive behavior and higher metastatic potential [3].

Accurate recognition of these tumors is essential, as their therapeutic management varies substantially depending on the subtype, histological grade, tumor size, and the presence of metastatic disease [4,5]. In clinical practice, diagnosis may be particularly challenging in older adults or polymorbid patients, in whom the tumor may be discovered incidentally or present with nonspecific gastrointestinal symptoms [6,7]. We report the case of an elderly woman diagnosed with a type 1 G-NET in the context of autoimmune gastritis, emphasizing the importance of individualized diagnostic and therapeutic strategies in older adults.

## Case presentation

An 84-year-old female patient with a history of systemic arterial hypertension presented with an eight-month history of progressive fatigue, unintentional weight loss, anorexia, and intermittent diarrhea. There was no family history of cancer, tuberculosis, or autoimmune/autoinflammatory diseases. On physical examination, she appeared malnourished. Initial testing showed a positive fecal occult blood test and significant microcytic hypochromic anemia, requiring transfusion of two units of packed red blood cells. She was admitted for further evaluation.

Laboratory testing upon hospital admission revealed persistent microcytic anemia with markedly low iron indices and transferrin saturation, consistent with iron-deficiency anemia. There was no laboratory evidence of hemolysis. Liver, renal, and electrolyte panels were within normal limits. Inflammatory markers were not elevated, and vitamin B12 levels were preserved. Serum gastrin was markedly elevated, whereas gastrin-releasing peptide remained within the normal range (Table 1).

**Table 1:** Laboratory parameters at hospital admission CRP: C-reactive protein; ESR: erythrocyte sedimentation rate; LDH: lactate dehydrogenase; MCV: mean corpuscular volume; ProGRP: gastrin-releasing peptide; HE4: human epididymis protein 4; IGRA: interferon gamma release assay; CA 125: cancer antigen 125

Relevant laboratory results at admission		
			Test	Result	Reference range (local)
Hemoglobin (pretransfusion)	6.8 g/dL	11.7-14.1 g/dL
Hemoglobin (posttransfusion at admission)	8.9 g/dL	11.7-14.1 g/dL
Mean corpuscular volume (MCV)	73.6 fL	73.0-91.0 fL
Serum iron	10.5 µg/dL	60-180 µg/dL
Ferritin	12 ng/mL	20-200 ng/mL
Transferrin saturation	2.15%	20-60%
Reticulocyte count	0.9%	0.5-1.5%
Haptoglobin	2.6 g/L	0.3-2.0 g/L
Lactate dehydrogenase (LDH)	173 IU/L	140-271 IU/L
Total bilirubin	0.57 mg/dL	0.3-1.0 mg/dL
Direct bilirubin	0.14 mg/dL	0.03-0.18 mg/dL
Indirect bilirubin	0.43 mg/dL	0.2-0.8 mg/dL
Direct antiglobulin test (Coombs)	Negative	-
Indirect antiglobulin test	Negative	-
C-reactive protein (CRP)	<5.0 mg/L	0.0-5.0 mg/L
Erythrocyte sedimentation rate (ESR)	25 mm/h	0.0-25.0 mm/h
Vitamin B12	378.0 pg/mL	180-914 pg/mL
Serum gastrin	920.0 pg/mL	13.0-115.0 pg/mL
Gastrin-releasing peptide (ProGRP)	38.0 pg/mL	0.0-66.3 pg/mL
Fecal occult blood	Positive	-
CA 125	10 U/mL	<35 U/mL
CA 19-9	12 U/mL	<37 U/mL
HE4	77.7	<104 (≥70 years)
IGRA (Quantiferon-TB)	Positive	-

**Figure 1:**
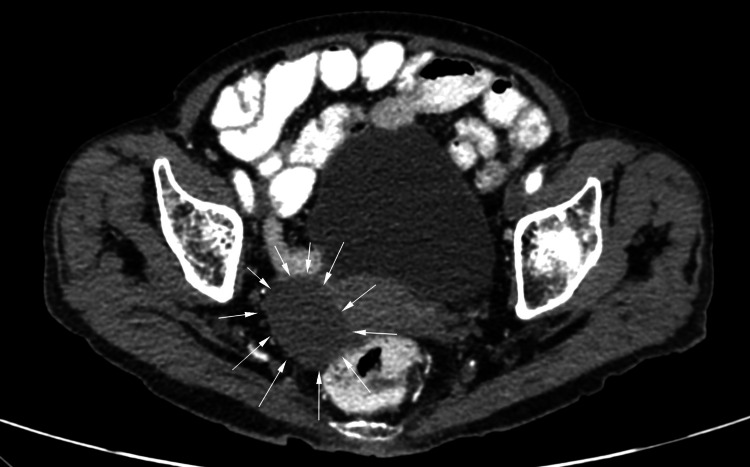
Abdominopelvic CT showing right ovarian cyst Well-defined cyst with regular borders and no local invasion

An abdominopelvic computed tomography performed as part of the diagnostic screening identified a right ovarian cyst with regular borders and no evidence of local invasion (Figure 1). Tumor markers, including cancer antigen 125 (CA-125), CA 19-9, and carcinoembryonic antigen (CEA), were within normal limits, and malignancy was ruled out by the gynecology team (Table 1).

A positive interferon-gamma release assay (IGRA) prompted evaluation for active tuberculosis, including chest radiography, thoracoabdominopelvic computed tomography, and cervical ultrasound, all of which yielded normal findings. Three induced sputum samples tested negative for acid-fast bacilli.

**Figure 2:**
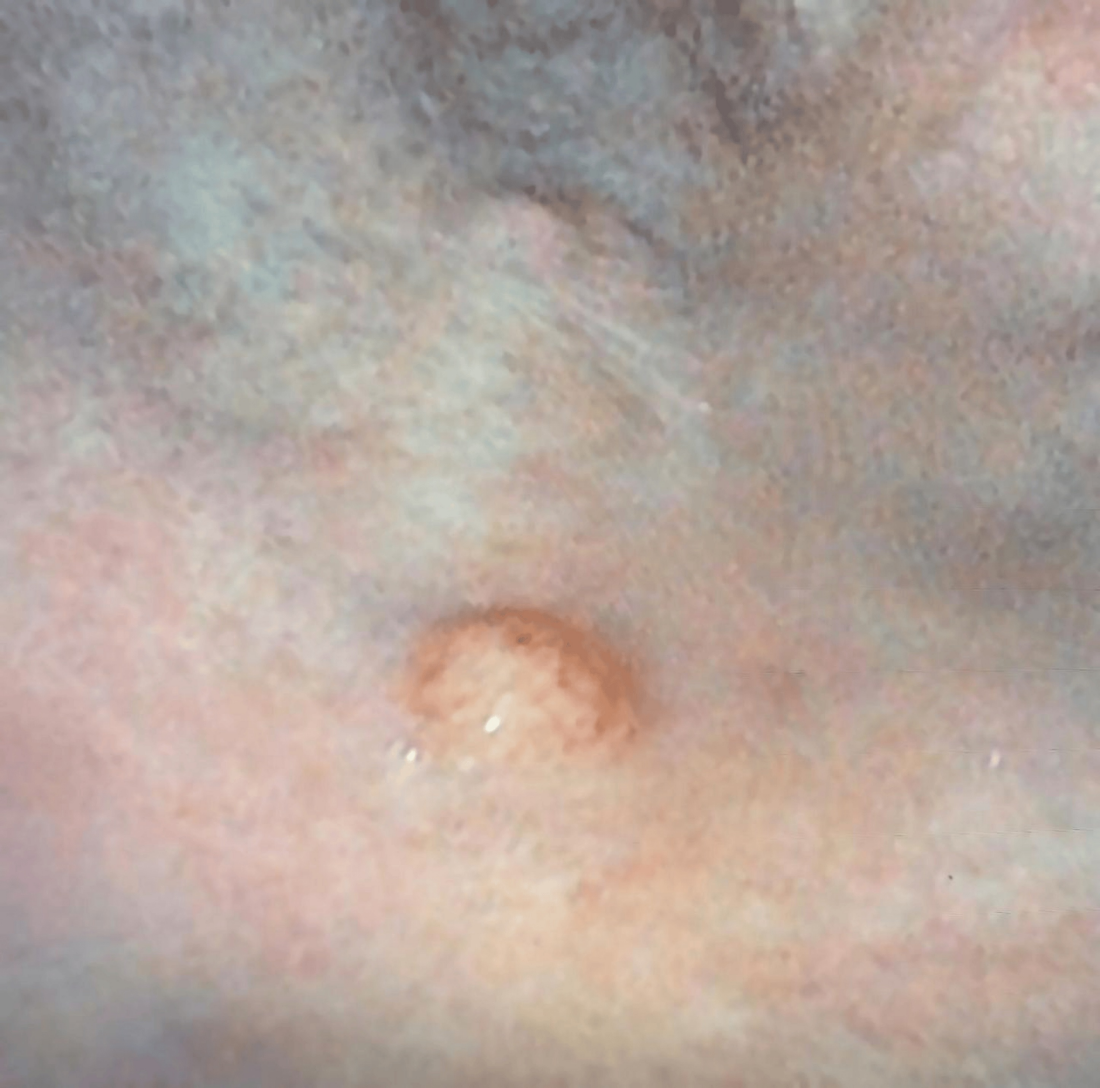
Endoscopic image of the gastric fundus showing a small, sessile, hypervascular polyp The lesion was found during routine esophagogastroduodenoscopy in an elderly patient with anemia. The surrounding mucosa shows signs of atrophy

**Figure 3:**
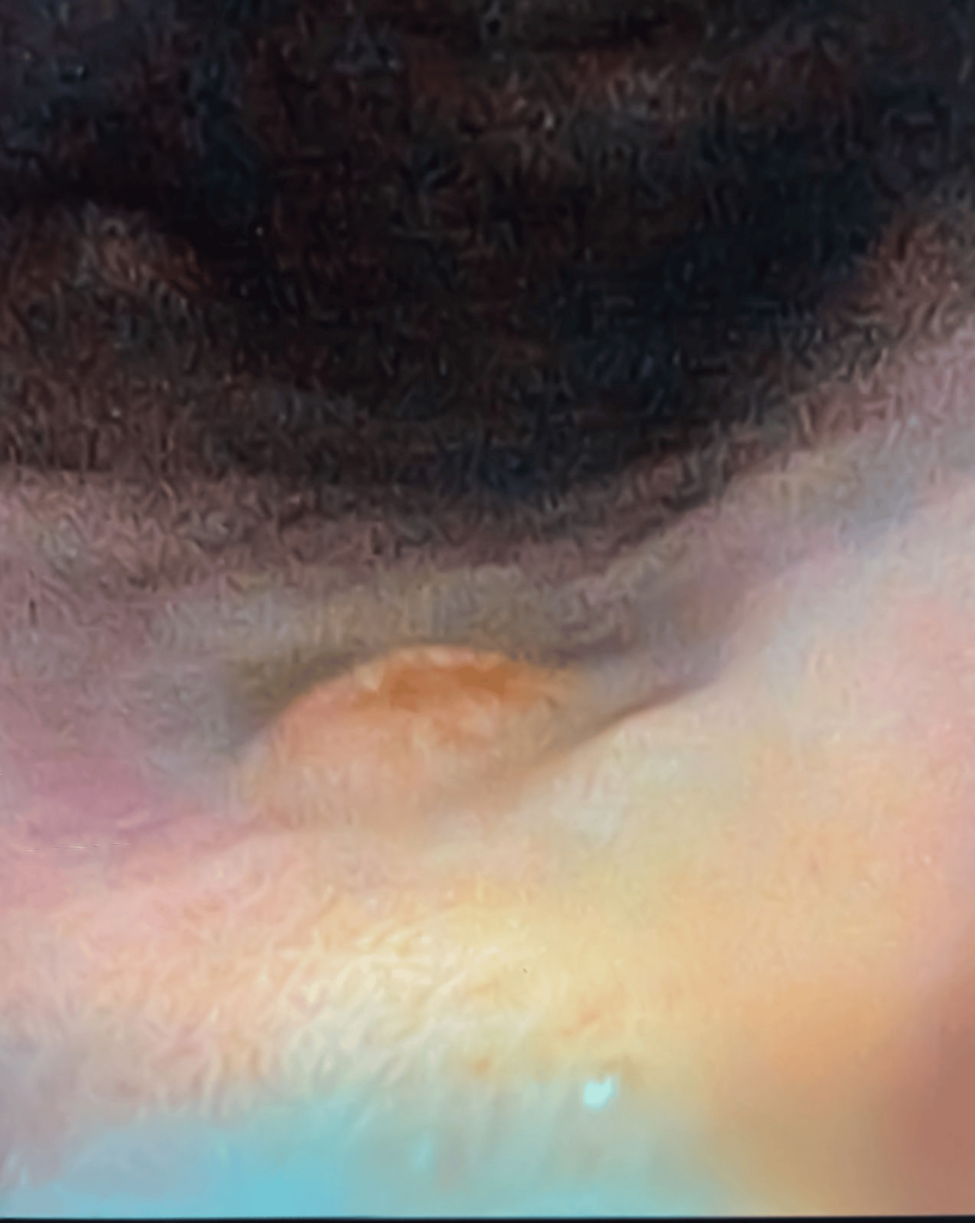
Closer endoscopic view of a hypervascular polyp in the gastric body The smooth and well-circumscribed appearance is characteristic of type 1 gastric neuroendocrine tumors

Upper gastrointestinal endoscopy revealed atrophic mucosa in the gastric fundus and body, along with multiple sessile polyps exhibiting increased vascularity (Figures 2, 3). Biopsies were taken. Colonoscopy ruled out colorectal pathology. The patient was not receiving proton pump inhibitors, which helped exclude drug-induced hypergastrinemia and supported autoimmune atrophic gastritis as the underlying cause.

**Figure 4:**
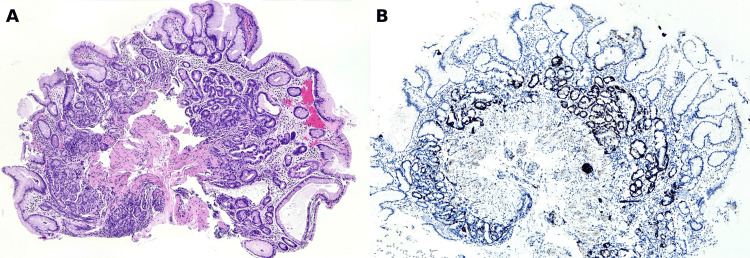
Hyperplasia of gastric enterochromaffin-like (ECL) cells (A) Gastric mucosa showing increased numbers of ECL cells distributed along the base of the fundic glands. (B) Immunohistochemistry for synaptophysin showing strong and diffuse cytoplasmic expression in the hyperplastic neuroendocrine cells, with a linear and focally nodular pattern

Histological evaluation of the gastric biopsies confirmed chronic atrophic gastritis with intestinal and pyloric metaplasia affecting the fundus and body. In several areas, there was linear and focally nodular hyperplasia of ECL cells, as illustrated in Figure 4A, with strong synaptophysin expression shown in Figure 4B. 

**Figure 5:**
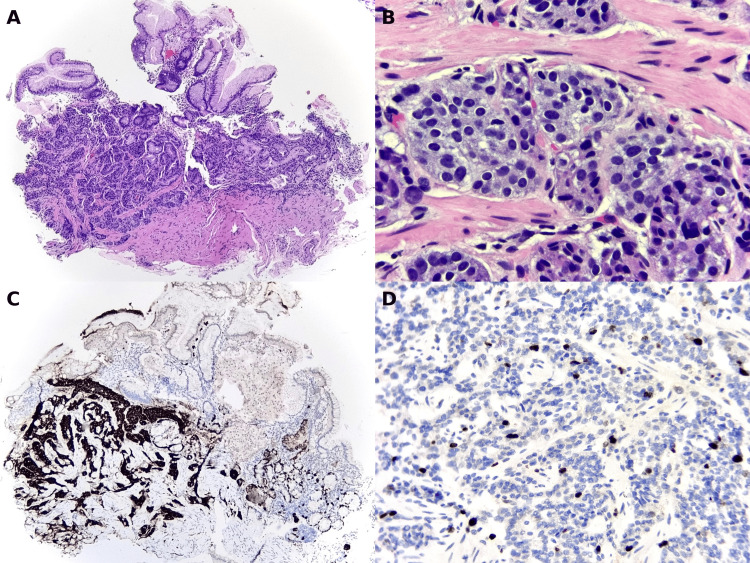
Gastric neuroendocrine tumor in endoscopic biopsy (A) Gastric mucosa showing a solid and trabecular proliferation of tumor cells infiltrating the mucosa and submucosa, with focal extension into the muscularis mucosae. (B) At higher magnification, the nests are composed of cells with round nuclei, finely granular “salt and pepper” chromatin (characteristic of neuroendocrine tumors), and scant eosinophilic cytoplasm. (C) Immunohistochemistry for chromogranin showing strong cytoplasmic expression in tumor cells. (D) Ki-67 immunostaining showing a proliferation index of approximately 5%

In other fragments, a well-differentiated neuroendocrine tumor was identified, composed of solid and trabecular nests of cells infiltrating the mucosa and submucosa, with focal extension into the muscularis mucosae (Figure 5A). At higher magnification (Figure 5B), the tumor cells exhibited round nuclei, finely granular “salt and pepper” chromatin, and scant eosinophilic cytoplasm. Immunohistochemistry was positive for chromogranin (Figure 5C) and synaptophysin, and the Ki-67 proliferation index was 5% (Figure 5D), consistent with a WHO grade II neuroendocrine tumor. *Helicobacter pylori* was not detected by a special stain.

The patient was evaluated by medical oncology, who considered surgical resection a viable option. Digestive surgery and oncogeriatrics subsequently performed a comprehensive assessment of functionality and life expectancy to support a personalized treatment strategy. Ultimately, given the patient’s comorbidities and tumor characteristics, surgery was deferred, and endoscopic surveillance was selected as the preferred management approach.

## Discussion

Type 1 G-NETs represent nearly 80% of gastric NETs and are strongly associated with autoimmune chronic atrophic gastritis [2,3]. The pathophysiology involves progressive loss of parietal cells, hypochlorhydria, and ECL cell hyperplasia secondary to chronic hypergastrinemia, eventually resulting in neoplasia [1,3]. These lesions are often small, multiple, and localized to the fundus and body, with low metastatic potential [5].

Accurate clinical and histological classification is critical. The WHO recommends the use of the Ki-67 index and mitotic count to categorize tumors as grade 1, 2, or 3, regardless of clinical subtype [4]. Diagnosis is supported by gastrin and gastrin-releasing peptide (GRP) levels, along with vitamin B12 as an indirect marker of the autoimmune context [3,6]. The most prevalent clinical condition associated with type 1 G-NETs is chronic autoimmune atrophic gastritis, which was also present in this patient.

This case illustrates a frail elderly patient with nonspecific symptoms and severe iron-deficiency anemia. Following the exclusion of infectious, colorectal, and gynecologic malignancies, endoscopic and histological findings, in conjunction with elevated gastrin levels, confirmed the diagnosis of type 1 G-NET associated with autoimmune gastritis.

Management is tailored based on size, grade, and recurrence. Most small, low-grade type 1 G-NETs can be followed with surveillance or local resection [5]. For larger or recurrent lesions, antrectomy or subtotal gastrectomy may be indicated [8]. In elderly or frail patients, a functional and life expectancy assessment is crucial to weigh treatment risks and benefits. Literature supports an individualized approach in such cases [9,10]. In the present case, given the lesion’s low grade, small size, and absence of metastasis, the multidisciplinary team opted for endoscopic surveillance as the most appropriate management strategy.

## Conclusions

This case highlights the importance of considering G-NETs in the differential diagnosis of nonspecific gastrointestinal symptoms and severe iron-deficiency anemia in elderly patients. Type 1 G-NETs, though generally indolent, require a systematic diagnostic approach involving endoscopy, histology, and biochemical markers. Management strategies should be tailored based on tumor characteristics, recurrence risk, and especially the patient’s functional status and comorbidities. Multidisciplinary evaluation is essential to ensure safe and appropriate treatment decisions in this population. This case underscores the importance of individualized care in geriatric oncology.
